# Lower Risks of Gastrointestinal Perforation and Intestinal Obstruction in Patients with Atypical Antipsychotics in Comparison with Typical Antipsychotics Based on Real-World Data from the MID-NET^®^ in Japan

**DOI:** 10.1007/s43441-023-00586-2

**Published:** 2023-10-29

**Authors:** Tomoaki Hasegawa, Sono Sawada, Tomoyuki Saito, Mei Kohama, Kazuhiro Kajiyama, Chieko Ishiguro, Takahiro Nonaka, Toshiyuki Okamura, Yukari Iwasaki, Takahiro Ueda, Toyotaka Iguchi, Naoya Horiuchi, Yoshiaki Uyama

**Affiliations:** 1 Office of Medical Informatics and Epidemiology, Pharmaceuticals and Medical Devices Agency, Kasumigaseki 3-3-2, Chiyoda-ku, Tokyo, 100-0013 Japan; 2 Office of Pharmacovigilance I, Pharmaceuticals and Medical Devices Agency, Tokyo, Japan; 3 Office of Pharmacovigilance II, Pharmaceuticals and Medical Devices Agency, Tokyo, Japan; 4 Present Address: IQVIA Solutions Japan K.K., Tokyo, Japan; 5 Present Address: Section of Clinical Epidemiology, Department of Data Science, Center for Clinical Sciences, National Center for Global Health and Medicine, Tokyo, Japan; 6 Present Address: Department of Health and Medical Innovation, Graduate School of Medicine, Osaka Metropolitan University, Osaka, Japan

**Keywords:** Atypical antipsychotics, Gastrointestinal perforation, Intestinal obstruction, Pharmaco-epidemiology, Real-world evidence

## Abstract

Intestinal perforation and obstruction are known to be one of the adverse events caused by antipsychotics; however, warning information on package inserts varies among antipsychotics. To investigate the risks of gastrointestinal perforation and intestinal obstruction in patients prescribed atypical antipsychotics compared with those in patients prescribed typical antipsychotics, a nested case–control study was conducted utilizing real-world data from the MID-NET^®^ medical information database in Japan. The study period spanned from January 1, 2009, to December 31, 2018. We found that the risks of gastrointestinal perforation and intestinal obstruction in patients prescribed atypical antipsychotics were significantly lower than those in patients prescribed typical antipsychotics (adjusted odds ratio, 0.48; 95% confidence interval, 0.29–0.80). This finding was supported with prolonged periods for the exposure definition in the sensitivity analyses. In addition, no major differences in the risks of atypical antipsychotics, such as risperidone, quetiapine, olanzapine, and aripiprazole, were identified in this study. The safety profile regarding the lower risks of gastrointestinal perforation and intestinal obstruction in patients prescribed atypical antipsychotics should be considered when choosing antipsychotics in clinical practice in terms of the proper use of such drugs.

**Supplementary Information:**

The online version contains supplementary material available at 10.1007/s43441-023-00586-2.

## Introduction

In March 2019, the risk of intestinal ulcer and perforation, in addition to intestinal obstruction, was added to the section “clinically significant adverse reactions” (CSARs) on the package insert (PI) of clozapine in Japan, based on accumulated spontaneous adverse drug reaction reports [1]. Conversely, ileus paralytic, which is generally considered less severe than intestinal ulcer and perforation, was mentioned as a gastrointestinal-related CSARs in the PIs of other atypical antipsychotics [2]. As the anticholinergic effects and safety risk profiles vary among antipsychotics [3–5], further investigations are important to quantitatively examine the risks of gastrointestinal perforation and intestinal obstruction in patients taking atypical antipsychotics.

Therefore, the Pharmaceuticals and Medical Devices Agency (PMDA) decided to conduct a pharmaco-epidemiological study to examine the risk of gastrointestinal perforation and intestinal obstruction, as an index of gastrointestinal dysfunction, in association with atypical antipsychotics.

## Methods

### Database

Real-world data from MID-NET^®^, a reliable and valuable database in Japan [6, 7], were used for analysis in this study because MID-NET^®^ stores electronic medical records, administrative claim data, and diagnosis procedure combination (DPC) data of over 6.05 million patients (as of December 2022) in cooperation with 10 healthcare organizations, including 23 university hospitals and regional core hospitals. The study period spanned from January 1, 2009, to December 31, 2018.

Utilizing MID-NET^®^ for this study was approved on October 30, 2019, through a discussion by the expert committee of MID-NET^®^ [8] and the actual data extraction from MID-NET^®^ for analysis was carried out on November 26, 2019. As this study was conducted as an official activity of the PMDA under the Pharmaceuticals and Medical Devices Agency Law (Articles 15–5–(c) and (f)), it was not subject to a review through an institutional review board [9, 10].

### Study Design

A nested case–control design rather than a cohort design was selected to examine a safety signal by antipsychotics more comprehensively and consider many situations, such as switching and/or concomitant use of antipsychotics and its treatment length, comorbidities, and other concomitant drugs, on the occurrence of gastrointestinal perforation and intestinal obstruction.

### Cohort

The primary cohort comprised patients who were prescribed atypical or typical antipsychotics during the study period, but excluded patients admitted for the treatment of gastrointestinal perforation or intestinal obstruction before t_0_ (the first prescription date of atypical or typical antipsychotics). Atypical antipsychotics investigated in this study were as follows: asenapine maleate, aripiprazole, aripiprazole hydrate, olanzapine, quetiapine fumarate, clozapine, paliperidone, paliperidone palmitate, brexpiprazole, blonanserin, perospirone hydrochloride hydrate, and risperidone (see supplementary Table S1 for the list of typical antipsychotics investigated in this study). For patient exclusion from the cohort, admission for the treatment of gastrointestinal perforation or intestinal obstruction was defined as a diagnosis of gastrointestinal perforation (excluding esophageal perforation or perforation of the appendix) with prescription of antibacterial drugs during hospital admission, or diagnosis of intestinal obstruction during hospital admission, respectively.

The follow-up period, a period to identify a case of gastrointestinal perforation or intestinal obstruction, started at t_0_ and ended at an earlier date according to the following: (1) the end date of the treatment period and (2) the date of occurrence of gastrointestinal perforation or intestinal obstruction. The treatment period comprised the prescription period (start date and duration of prescription) with a 90-day gap for an antipsychotic every 4-week continuous infusion, a 60-day gap for an antipsychotic every 2-week continuous infusion, and a 30-day gap for other antipsychotics (see Supplementary Table S1 for details of the gap period for each antipsychotic drug).

### Case and Control Definition

A case of gastrointestinal perforation or intestinal obstruction was identified in patients with a medical record of at least 30 days before t_0_. The outcome definitions of gastrointestinal perforation and intestinal obstruction used in this study were validated utilizing MID-NET^®^ data [11]. Specifically, cases of gastrointestinal perforation were counted when all the following criteria were met during the follow-up period: (1) diagnosis of gastrointestinal perforation (but not esophageal perforation or perforation of the appendix) during admission, (2) prescriptions of antibacterial drugs during admission, (3) examination of gastric intubation or gastrointestinal surgery during admission, and (4) examination or review of computed tomography in the period from 1 day before admission to the end date of admission. Similarly, cases of intestinal obstruction were counted when all the following criteria were met during the follow-up period: (1) diagnosis of intestinal obstruction during admission, (2) no surgery for intestinal obstruction during admission, and (3) examination or review using computed tomography/radiography in the period from 1 day before admission to the end date of admission. The index date of the case was the earliest date of admission for gastrointestinal perforation or intestinal obstruction as defined above. Patients whose index date was the same day as t_0_ were excluded.

For each case, controls (maximum 4) were selected by risk-set sampling from patients without the event of admission for the treatment of gastrointestinal perforation and intestinal obstruction, who had a medical record at least 30 days before t_0_ and were matched with a case based on the following variables: sex, age (± 5 years), healthcare organizations and calendar year at the index date (the admission date in one case), and number of admissions triggered by mental disorders (ICD-10 codes starting with “F”) from t_0_ to the index date. The index date of the controls was selected such that the follow-up time was equal to that of the cases.

### Exposure Definition

Patients treated with atypical or typical antipsychotics on the day before the index date were categorized into atypical or typical antipsychotic groups, respectively. The period to determine an exposure group was set based on the period of each prescription (start date and duration of prescription) with a gap period for each antipsychotic (see Supplementary Table S1 for details of the gap period for each antipsychotic). Patients who were concomitantly prescribed with both atypical and typical antipsychotics on the day before the index date were separately categorized into the different group (concomitant use of typical and atypical antipsychotics). Patients who received monotherapy with each atypical antipsychotic were also separately categorized into groups based on each active ingredient of antipsychotics.

Different definitions of exposure were used in the sensitivity analysis. Specifically, a timing to determine the exposure group (atypical or typical antipsychotic) was changed from “1 day before the index date” to “from 7 to 1 day before”, “14 to 1 day before”, “30 to 1 day before”, or “60 to 1 day before” the index date.

### Statistical Analysis

Patient background data, including matching factors and other relevant patient characteristics, were tabulated. To compare the risk of atypical antipsychotics with typical antipsychotics, conditional logistic regression analysis considering matching factors was conducted to estimate crude odds ratio (OR) and adjusted odds ratio (aOR) with adjustment for the following factors: (1) number of prescribed active ingredients of antipsychotics from t_0_ to 1 day before the index date, (2) category of average daily prescribed dose converted as a dose of chlorpromazine from t_0_ to 1 day before the index date (< 50 mg/day, 50 mg/day ≤  < 250 mg/day, 250 mg/day ≤  < 450 mg/day, 450 mg/day ≤) [12, 13], (3) concomitant drugs during 30 days before the index date (laxatives, NSAIDs, proton pump inhibitors, opioid analgesics, antineoplastics or immunosuppressants, belladonna alkaloid, psychotropics (including sleeping drugs, tranquilizers, anxiolytics, tricyclic antidepressants), anticholinergics), and (4) history of abdominal surgery before the index date. SAS version 9.4 (SAS Institute, Cary, NC, USA) was used for all the analyses.

## Results

### Cohort

**Figure 1:**
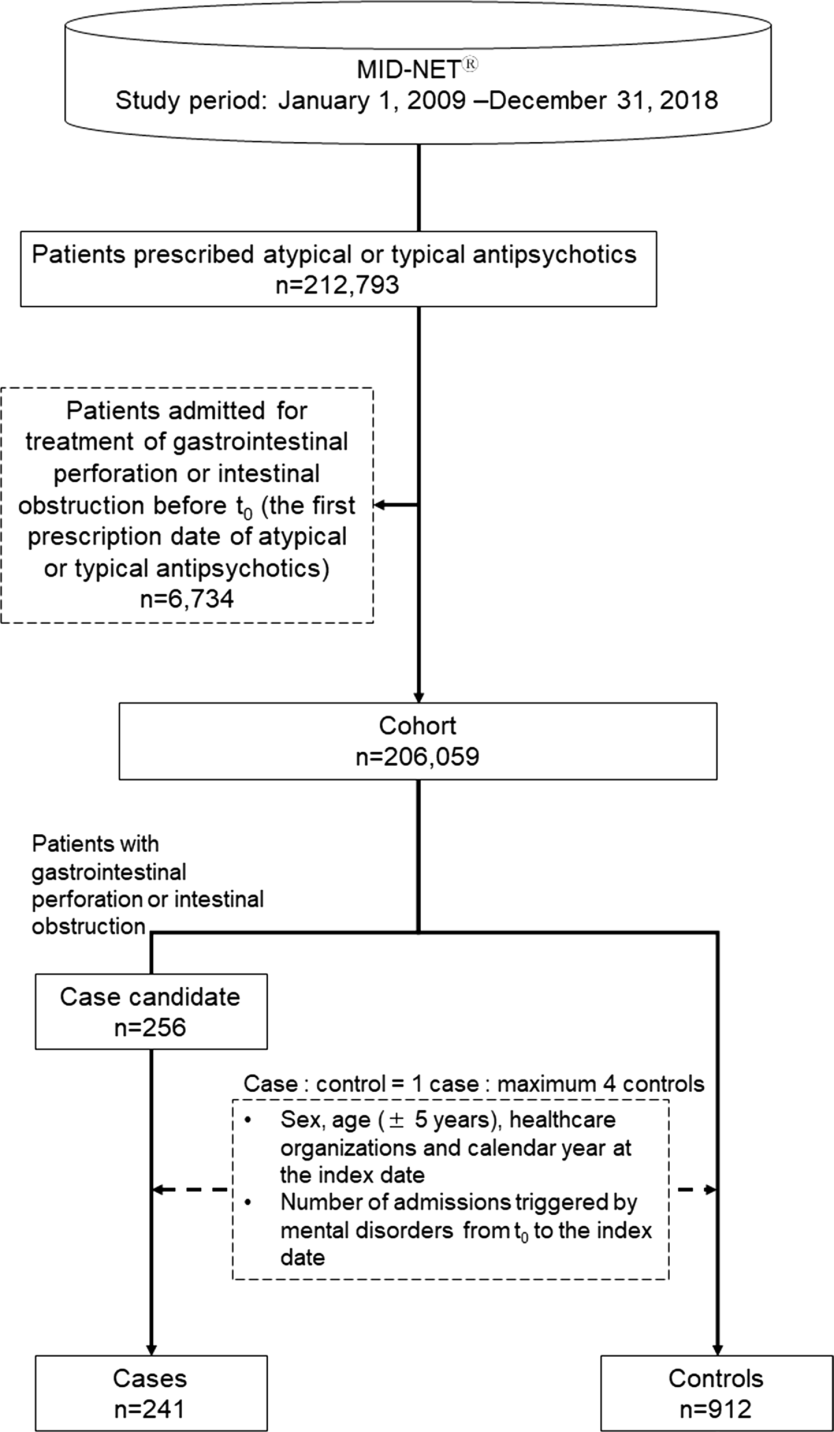
Study flowchart for patient selection.

Of 212,793 patients prescribed atypical or typical antipsychotics, 206,059 were included in the cohort after applying the exclusion criteria. In total, 241 cases and 912 controls were identified from the cohort for analysis (Fig. 1).

### Patient Characteristics

Table 1 shows the characteristics of the patients in this study, with similar distributions of matching variables between cases and controls. Some differences between cases and controls were found for non-matching variables, such as concomitant drugs and other medical histories; however, these factors were adjusted to calculate the aOR in the analysis.

**Table 1:** Characteristics of patients in this study

	Cases n = 241	Controls n = 912	Standardized difference
Sex^a^			
Female	132 (54.8%)	497 (54.5%)	0.006
Male	109 (45.2%)	415 (45.5%)	0.006
Median age (1Q-3Q)^a^	66.0 (56.0–75.0)	67.0 (57.0–75.0)	–
Calendar Year^a^			
2009–2011	55 (22.8%)	213 (23.4%)	0.013
2012–2014	96 (39.8%)	366 (40.1%)	0.006
2015–2017	67 (27.8%)	255 (28.0%)	0.004
2018–2019	23 (9.5%)	78 (8.6%)	0.035
Number of admissions due to psychiatric disease^b^			
No admissions	231 < (95.9% <) ^f^	902 < (98.9% <) ^f^	< 0.1 ^f^
1 or 2 times	< 10 (< 4.1%) ^f^	< 10 (< 1.1%) ^f^	< 0.1 ^f^
Median number of prescribed antipsychotics (1Q-3Q)^c^	1.0 (1.0–1.0)	1.0 (1.0–1.0)	–
Median of prescription days of antipsychotics during the past admission (1Q-3Q) before the index date^c^	2.0 (0.0–16.0)	0.0 (0.0–12.0)	–
Days from t_0_ to the index date			
Median (1Q-3Q)	34.0 (14.0–85.0)	32.0 (14.0–78.0)	–
≤ 90 days	182 (75.5%)	709 (77.7%)	0.053
91 days ≤ and ≤ 365 days	38 (15.8%)	128 (14.0%)	0.049
366 days <	21 (8.7%)	75 (8.2%)	0.018
Total prescribed doses (mg) converted as a dose of chlorpromazine^c^			
Median (1Q-3Q)	3,181.8 (1200.0–9000.0)	3300.0 (1241.7–9500.0)	–
Average daily prescribed doses (mg/days) converted as a dose of chlorpromazine^c^			
< 50	47 (19.5%)	183 (20.1%)	0.014
50 ≤ and < 250	158 (65.6%)	541 (59.3%)	0.129
250 ≤ and < 450	25 (10.4%)	88 (9.6%)	0.024
450 <	11 (4.6%)	100 (11.0%)	0.241
Concomitant drugs^d^			
Laxatives	172 (71.4%)	442 (48.5%)	0.481
NSAIDs	142 (58.9%)	400 (43.9%)	0.305
Proton pump inhibitors	114 (47.3%)	322 (35.3%)	0.245
Opioid analgesics	106 (44.0%)	138 (15.1%)	0.666
Antineoplastics or immunosuppressants	106 (44.0%)	213 (23.4%)	0.447
Belladonna alkaloid	38 (15.8%)	61 (6.7%)	0.291
Aspirin	17 (7.1%)	74 (8.1%)	0.040
Urinary antispasmodics	< 10 (< 4.1%) ^f^	24 (2.6%)	< 0.1^f^
Psychotropics (including sleeping drugs, tranquilizers, anxiolytics, tricyclic antidepressants)	< 10 (< 4.1%) ^f^	62 (6.8%)	< 0.2^f^
Anticholinergics	< 10 (< 4.1%) ^f^	58 (6.4%)	< 0.25^f^
Alpha-glucosidase inhibitors	< 10 (< 4.1%) ^f^	15 (1.6%)	< 0.1^f^
Polystyrene sulfonate	< 10 (< 4.1%) ^f^	11 (1.2%)	< 0.1^f^
Other medical histories^e^			
Diabetes	168 (69.7%)	597 (65.5%)	0.091
Scleroderma	< 10 (< 4.1%) ^f^	23 (2.5%)	< 0.1^f^
Abdominal surgery	38 (15.8%)	63 (6.9%)	0.282

1Q, 1st quarter; 3Q, 3rd quarter

^a^At the index date

^b^from t_0_ to the index date

^c^from t_0_ to 1 day before the index date

^d^during 30 days before the index date

^e^up to 1 day before the index date

^f^when the value was < 10, it was presented as an aggregated value based on the MID-NET^®^ publication rule

### Risk Comparison of Gastrointestinal Perforation and Intestinal Obstruction Between Atypical and Typical Antipsychotics

In comparing the risks of gastrointestinal perforation and intestinal obstruction by atypical antipsychotics with those by typical antipsychotics, aOR was 0.48 (95% confidence interval (CI), 0.29–0.80) indicating that the risk was significantly lower in atypical antipsychotics (Table 2). Lower aOR was still observed in patients with concomitant use of typical and atypical antipsychotics, although the upper range of the 95% CI exceeded 1.0 (aOR, 0.51; 95% CI, 0.23–1.16). Regarding the risk of a particular drug, OR (95% CI) for patients with monotherapy of risperidone, quetiapine, olanzapine, and aripiprazole was 0.35 (0.18–0.69), 0.30 (0.15–0.59), 0.40 (0.17–0.97), and 0.17 (0.05–0.58), respectively. For asenapine, paliperidone, blonanserin, and perospirone, the OR was not calculated because no cases were found in this study. In addition, no patients with the other atypical antipsychotics (i.e., clozapine and brexpiprazole) were observed in this study.

**Table 2:** Risk comparison of gastrointestinal perforation and intestinal obstruction between atypical and typical antipsychotics

	Cases	Controls	OR^a^ (95% CI)	aOR^a,b^ (95% CI)
Typical antipsychotics only	191	554	1.00 (reference)	1.00 (reference)
Atypical antipsychotics only	35	280	0.28 (0.19–0.44)	0.48 (0.29–0.80)
Concomitant use of typical and atypical antipsychotics	15	78	0.48 (0.26–0.89)	0.51 (0.23–1.16)

OR, crude odds ratio: aOR, adjusted odds ratio: CI, confidence interval

Categorization was based on types of antipsychotics on a day before the index date (see “METHODS”)

^a^Estimated using a conditional logistic regression model

^b^Variables for adjustment: number of prescribed antipsychotics from t_0_ to 1 day before the index date, and a category of average daily prescribed doses converted as a dose of chlorpromazine from t_0_ to 1 day before the index date, concomitant drugs during 30 days before the index date, history of abdominal surgery before the index date

As shown in Table 3, as the results of the sensitivity analysis, even when prolonging the period to determine the exposure group (atypical or typical antipsychotics), the risk of gastrointestinal perforation and intestinal obstruction was still lower in atypical antipsychotics than those in typical antipsychotics. For example, in case of atypical antipsychotics only, aOR (95% CI) was 0.49 (0.30–0.80) for a 7-day period (from 7 to 1 day before the index date), 0.48 (0.29–0.80) for a 14-day period (from 14 to 1 day before the index date), 0.52 (0.31–0.86) for a 30-day period (from 30 to 1 day before the index date), and 0.53 (0.32–0.88) for a 60-day period (from 60 to 1 day before the index date).

**Table 3:** Results of sensitivity analysis regarding the risk comparison of gastrointestinal perforation and intestinal obstruction between atypical and typical antipsychotics on the different exposure definitions

A timing to confirm the type of antipsychotics	Exposure group	Cases	Controls	OR^a^ (95% CI)	aOR^a,b^ (95% CI)
From 7 to 1 day before the index date (7-day period)	Typical antipsychotics only	191	553	1.00 (reference)	1.00 (reference)
	Atypical antipsychotics only	35	278	0.29 (0.19–0.44)	0.49 (0.30–0.80)
	Concomitant use of typical and atypical antipsychotics	15	81	0.46 (0.25–0.85)	0.47 (0.21–1.04)
From 14 to 1 day before the index date (14-day period)	Typical antipsychotics only	190	553	1.00 (reference)	1.00 (reference)
	Atypical antipsychotics only	34	273	0.28 (0.18–0.43)	0.48 (0.29–0.80)
	Concomitant use of typical and atypical antipsychotics	17	86	0.50 (0.28–0.90)	0.55 (0.26–1.17)
From 30 to 1 day before the index date (30-day period)	Typical antipsychotics only	187	550	1.00 (reference)	1.00 (reference)
	Atypical antipsychotics only	34	266	0.29 (0.19–0.45)	0.52 (0.31–0.86)
	Concomitant use of typical and atypical antipsychotics	20	96	0.52 (0.30–0.90)	0.45 (0.21–0.95)
From 60 to 1 day before the index date (60-day period)	Typical antipsychotics only	185	549	1.00 (reference)	1.00 (reference)
	Atypical antipsychotics only	33	260	0.29 (0.19–0.45)	0.53 (0.32–0.88)
	Concomitant use of typical and atypical antipsychotics	23	103	0.57 (0.34–0.95)	0.48 (0.23–1.00)

OR, crude odds ratio: aOR, adjusted odds ratio: CI, confidence interval

^a^Estimated using a conditional logistic regression model

^b^Variables for adjustment: number of prescribed antipsychotics from t_0_ to 1 day before the index date, and a category of average daily prescribed doses converted as a dose of chlorpromazine from t_0_ to 1 day before the index date, concomitant drugs during 30 days before the index date, history of abdominal surgery before the index date

## Discussion

The results of this study indicated that the risks of gastrointestinal perforation and intestinal obstruction in patients prescribed atypical antipsychotics were significantly lower than those in patients prescribed typical antipsychotics. This finding was supported with prolonged periods for the exposure definition in the sensitivity analyses. It has been reported that the use of typical antipsychotics (e.g., haloperidol, pimozide, and fluphenazine), clozapine, and anticholinergic drugs increases the risk of ileus through antagonistic effects on muscarinic receptors [14] and the risk of intestinal obstruction by clozapine may be higher than that by other standard antipsychotics [15]. The lower risk of gastrointestinal dysfunction in patients prescribed atypical antipsychotics in this study may be due to fewer anticholinergic (antimuscarinic) effects in comparison with typical antipsychotics, although the anticholinergic effects may differ among antipsychotic drugs [3–5]. Particularly, the lack of patients with clozapine, categorized as an atypical antipsychotic in this study and known to have a higher risk of gastrointestinal dysfunction, may contribute to the results of this study. The lack of patients taking clozapine could be due to strict regulations in prescribing this drug, called the Clozaril Patient Monitoring Service (CPMS), which limits medical institutions that can prescribe clozapine for careful monitoring of serious adverse events such as agranulocytosis [16]. Conversely, the risk differences among atypical antipsychotics could not be sufficiently evaluated in this study because of the limited number of patients or no patients with monotherapy, although the ORs for patients receiving monotherapy with risperidone, quetiapine, olanzapine, and aripiprazole were consistently lower than 1.00, suggesting no major differences in the risk of gastrointestinal dysfunction among these antipsychotics. The absence of patients taking brexpiprazole in this study could have resulted from the shorter study period (only 8 months) for this drug, which was marketed in Japan in April 2018.

The strength of this study was the use of the validated outcome definitions for gastrointestinal perforation and intestinal obstruction [11] as well as utilizing real-world data from the MID-NET^®^, a reliable database [6, 7]. However, as a limitation, we targeted patients who were prescribed atypical or typical antipsychotics during the study period regardless of their disease. Thus, the results may have been affected by other potential confounders, such as disease severity and concomitant drugs, which were not considered in this study.

The PMDA conducted a safety assessment of the risk of gastrointestinal dysfunction associated with atypical antipsychotics based on the study results and other information, including case reports and related literature, and concluded that no additional safety measures for atypical antipsychotics are required at present.

## Conclusion

The risks of gastrointestinal perforation and intestinal obstruction in patients prescribed atypical antipsychotics were significantly lower than those in patients prescribed typical antipsychotics. This should be considered in the choice of antipsychotics in clinical practice in terms of the proper use of such drugs.

### Supplementary Information

Below is the link to the electronic supplementary material.

Supplementary file1 (DOCX 21 kb)

## References

[1] Pharmaceuticals and Medical Devices Agency, Summary of Investigation Results, Clozapine 2019 [Available from: https://www.pmda.go.jp/files/000228667.pdf].

[2] Pharmaceuticals and Medical Devices Agency, Information search for new drugs, such as review reports, package inserts, and other related infromation [in Japanese] 2022 [Available from: https://www.pmda.go.jp/PmdaSearch/iyakuSearch/].

[3] Huhn M, Nikolakopoulou A, Schneider-Thoma J, Krause M, Samara M, Peter N (2019). Comparative efficacy and tolerability of 32 oral antipsychotics for the acute treatment of adults with multi-episode schizophrenia: a systematic review and network meta-analysis. Lancet.

[4] Solmi M, Murru A, Pacchiarotti I, Undurraga J, Veronese N, Fornaro M (2017). Safety, tolerability, and risks associated with first- and second-generation antipsychotics: a state-of-the-art clinical review. Ther Clin Risk Manag.

[5] Inada K, Oshibuchi H, Ishigooka J, Nishimura K (2018). Analysis of clozapine use and safety by using comprehensive national data from the Japanese clozapine patient monitoring service. J Clin Psychopharmacol.

[6] Yamaguchi M, Inomata S, Harada S, Matsuzaki Y, Kawaguchi M, Ujibe M (2019). Establishment of the MID-NET^®^ medical information database network as a reliable and valuable database for drug safety assessments in Japan. Pharmacoepidemiol Drug Saf.

[7] Yamada K, Itoh M, Fujimura Y, Kimura M, Murata K, Nakashima N (2019). The utilization and challenges of Japan’s MID-NET^®^ medical information database network in post-marketing drug safety assessments: a summary of pilot pharmacoepidemiological studies. Pharmacoepidemiol Drug Saf.

[8] Pharmaceuticals and Medical Devices Agency. Information for studies approved by the expert committee of MID-NET^®^ [in Japanese]: Pharmaceuticals and Medical Devices Agency; [Available from: https://www.pmda.go.jp/safety/mid-net/0010.html].

[9] Act on the Pharmaceuticals and Medical Devices Agency (Act No.192 of 2002). [in Japanese] [Available from: https://elaws.e-gov.go.jp/document?lawid=414AC0000000192].

[10] Pharmaceuticals and Medical Devices Agency, Pharmacoepidemiological studies for drug safety assessment under MIHARI framework [in Japanese] [Available from: https://www.pmda.go.jp/safety/surveillance-analysis/0045.html].

[11] Research group on characterization and methods of extraction and analysis of MID-NET data, under the research on regulatory science of pharmaceuticals and medical devices supported by Japan Agency for Medical Research and Development, Grant No. 17mk0101088h0001: FY2017-FY2019 2019 [Available from: https://amedfind.amed.go.jp/amed/search/task_search_details.html].

[12] The Japanese Society of Psychiatric Rating Scales, Equivalent conversion table for psychotropic drugs 2017 2017 [Available from: http://jsprs.org/toukakansan/2017ver/].

[13] Inada T, Inagaki A (2015). Psychotropic dose equivalence in Japan. Psychiatry Clin Neurosci.

[14] Nielsen J, Meyer JM (2012). Risk factors for ileus in patients with schizophrenia. Schizophr Bull.

[15] Stroup TS, Gerhard T, Crystal S, Huang C, Olfson M (2016). Comparative effectiveness of clozapine and standard antipsychotic treatment in adults with schizophrenia. Am J Psychiatry.

[16] The Expert Committee for Clozaril Patient Monitoring Service [Available from: https://www.clozaril-tekisei.jp/].

