# Supplementary material for: Lower Risks of Gastrointestinal Perforation and Intestinal Obstruction in Patients with Atypical Antipsychotics in Comparison with Typical Antipsychotics Based on Real-World Data from the MID-NET® in Japan
Source: Ther Innov Regul Sci. 2023 Oct 29;58(1):192–9. doi: 10.1007/s43441-023-00586-2 (PMC10764367; doi:10.1007/s43441-023-00586-2)
Supplement: Supplementary file 1 — Supplementary file1 (DOCX 21 kb) [file 43441_2023_586_MOESM1_ESM.docx]

**Article title:**

Lower risks of gastrointestinal perforation and intestinal obstruction in patients with atypical antipsychotics in comparison with typical antipsychotics based on real-world data from the MID-NET^®^ in Japan

**Journal name:**

Therapeutic Innovation & Regulatory Science

**Authors:**

Tomoaki Hasegawa^1^, Sono Sawada^1^, Tomoyuki Saito^1^, Mei Kohama^1^, Kazuhiro Kajiyama^1^, Chieko Ishiguro^1^, Takahiro Nonaka^1^, Toshiyuki Okamura^2^, Yukari Iwasaki^2^, Takahiro Ueda^2^, Toyotaka Iguchi^3^, Naoya Horiuchi^2^ and Yoshiaki Uyama^1^

**Affiliation:**

1: Office of Medical Informatics and Epidemiology, Pharmaceuticals and Medical Devices Agency

2: Office of Pharmacovigilance I, Pharmaceuticals and Medical Devices Agency

3: Office of Pharmacovigilance II, Pharmaceuticals and Medical Devices Agency

**Corresponding author information:**

Name: Yoshiaki Uyama

E-mail address: uyama-yoshiaki@pmda.go.jp

**Supplementary Table S1. List of antipsychotics and its gap periods in this study**

1. Atypical antipsychotics

| Generic names | Gap period (day) |
| --- | --- |
| Asenapine maleate | 30 |
| Aripiprazole and aripiprazole hydrate | 90 (every 4-week continuous infusion)  or 30 (others) |
| Olanzapine | 30 |
| Quetiapine fumarate | 30 |
| Clozapine* | 30 |
| Paliperidone and paliperidone palmitate | 90 (every 4-week continuous infusion)  or 30 (others) |
| Brexpiprazole* | 30 |
| Blonanserin | 30 |
| Perospirone hydrochloride hydrate | 30 |
| Risperidone | 60 (every 2-week continuous infusion)  or 30 (others) |

*Antipsychotics without prescription observed in this study

1. Typical antipsychotics

| Generic names | Gap period (day) |
| --- | --- |
| Oxypertine* | 30 |
| Clocapramine hydrochloride hydrate | 30 |
| Chlorpromazine hydrochloride, chlorpromazine phenolphthalinate, chlorpromazine hibenzate, and the combination product of chlorpromazine hydrochloride with phenobarbital and promethazine hydrochloride | 30 |
| Spiperone* | 30 |
| Sultopride hydrochloride | 30 |
| Sulpiride | 30 |
| Zotepine | 30 |
| Timiperone* | 30 |
| Nemonapride | 30 |
| Haloperidol and haloperidol decanoate | 90 (every 4-week continuous infusion)  or 30 (others) |
| Pipamperone hydrochloride | 30 |
| Pimozide | 30 |
| Fluphenazine decanoate, fluphenazine enanthate, and fluphenazine maleate | 90 (every 4-week continuous infusion)  or 30 (others) |
| Prochlorperazine maleate and prochlorperazine mesilate | 30 |
| Propericiazine | 30 |
| Bromperidol | 30 |
| Perphenazine, perphenazine hydrochloride, perphenazine maleate, and perphenazine fendizoate | 30 |
| Mosapramine hydrochloride* | 30 |
| Levomepromazine hydrochloride and levomepromazine maleate | 30 |

*Antipsychotics without prescription observed in this study
